# Low Dynamics, High Longevity and Persistence of Sessile Structural Species Dwelling on Mediterranean Coralligenous Outcrops

**DOI:** 10.1371/journal.pone.0023744

**Published:** 2011-08-24

**Authors:** Núria Teixidó, Joaquim Garrabou, Jean-George Harmelin

**Affiliations:** 1 Ecology Department, Biology Faculty, University of Barcelona, Barcelona, Spain; 2 Institute of Marine Sciences (ICM-CSIC), Barcelona, Spain; 3 Centre d'Océanologie de Marseille, Université de la Mediterranée, CNRS - UMR 6540 DIMAR, Marseille, France; National Institute of Water & Atmospheric Research, New Zealand

## Abstract

There is still limited understanding of the processes underlying benthic species dynamics in marine coastal habitats, which are of disproportionate importance in terms of productivity and biodiversity. The life-history traits of long-lived benthic species in these habitats are particularly poorly documented. In this study, we assessed decadal patterns of population dynamics for ten sponge and anthozoan species that play key structural roles in coralligenous outcrops (∼25 m depth) in two areas of the NW Mediterranean Sea. This study was based on examination of a unique long-term photographic series, which allowed analysis of population dynamics over extensive spatial and time spans for the very first time. Specifically, 671 individuals were censused annually over periods of 25-, 15-, and 5-years. This long-term study quantitatively revealed a common life-history pattern among the ten studied species, despite the fact they present different growth forms. Low mortality rates (3.4% yr^−1^ for all species combined) and infrequent recruitment events (mean value of 3.1±0.5 SE recruits yr^−1^) provided only a very small fraction of the new colonies required to maintain population sizes. Overall, annual mortality and recruitment rates did not differ significantly among years; however, some species displayed important mortality events and recruitment pulses, indicating variability among species. Based on the growth rates of these 10 species, we projected their longevity and, obtained a mean estimated age of 25–200 years. Finally, the low to moderate turnover rates (mean value 0.80% yr^−1^) observed among the coralligenous species were in agreement with their low dynamics and persistence. These results offer solid baseline data and reveal that these habitats are among the most vulnerable to the current increases of anthropogenic disturbances.

## Introduction

Ecosystems worldwide are changing as a result of a number of natural drivers and the effects of myriad anthropogenic activities [1], [2], [3]. The increases in the impacts of these activities (e.g. overexploitation, habitat modification, effects of climate change) raise concerns about the capacity of ecosystems to absorb multiple disturbances occurring over short time periods [4], [5], [6]. An important goal of ecology and conservation research is to obtain a precise quantitative understanding of community dynamics by quantifying rates of change and evaluating the ecological mechanisms behind the observed changes, which will ultimately allow us to develop our capacity to predict longer-term and larger-scale shifts.

Coastal marine systems are a main focus of attention because they harbor high biological diversity, are among the most productive systems in the world, and present high anthropogenic interaction levels [7], [3]. Biodiversity losses in coastal marine ecosystems are increasingly impairing the ocean's capacity to provide ecosystem services, such as food supplies, maintaining water quality, recreation and tourism [8], [9], [10]. In the Mediterranean Sea, coralligenous outcrops are of special concern, as they represent one of the most important hotspot for species diversity in the Mediterranean Sea (harboring around 20% of Mediterranean species), are of great structural complexity and are dominated by long-lived species [11], [12]–[13]. Coralligenous outcrops are increasingly suffering impacts of a range of anthropogenic disturbances (e.g., fishing activities, pollution, increases of sedimentation, introduction of invasive species, climate change) [14], [15], [16], [17], [18]

A key factor for understanding and predicting responses of marine benthic species to variations is measurement of their vital rates e.g. reproduction, recruitment, growth, and mortality [19], [20]. In long-lived benthic organisms, detailed long-term studies following the fate of species can be considered the only reliable way to uncover general patterns. First, populations of slow-growing, long-lived sessile species do not often undergo marked declines, and populations with little or no regeneration capacity are projected to survive for decades or even centuries [21], [22], [23]. Second, crucial events occur rarely (e.g. severe disturbances or exceptional recruitment), and thus, detecting their effects on populations requires long-term records [24], [25], [26]. In contrast to terrestrial plant systems, such data are scarce for marine benthic assemblages, and little empirical work has been done on the population dynamics of long-lived marine sessile species.

The decadal trajectory of vital rates of species dwelling in coralligenous Mediterranean outcrops remains poorly understood, mainly due to the lack of available data from long-term datasets. Even basic parameters, such as survivorship, growth, age at first reproduction, and longevity, are unknown for the majority of coralligenous species. Therefore, a detailed understanding of the life cycle of such species and the long-term temporal variation in their vital rates is crucial, not only to comprehend the mechanisms underlying their dynamics but also for the effective management and conservation of these ecosystems.

**Figure 1:**
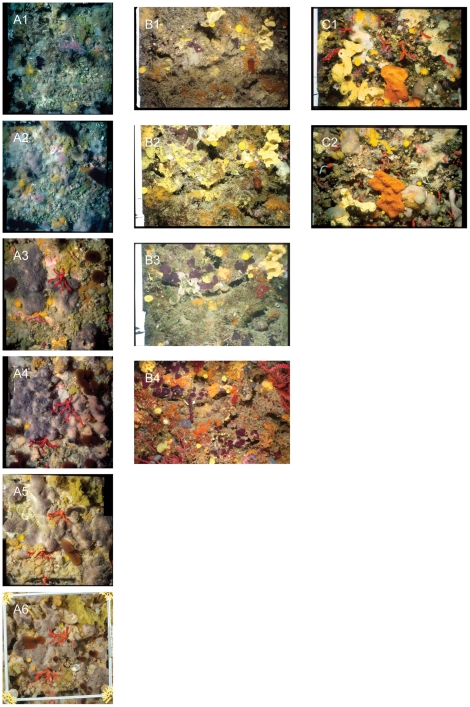
Example of the frames analyzed from the three photographic series over 25-, 15- and 5- years. A) The 25- year series at Riou Archipelago; dates A1: 23/01/1981, A2: 31/10/1986, A3: 25/11/1991, A4: 23/05/1996, A5: 06/03/2001, A6: 01/11/2006. B) The 15- year series at Pota de Llop; dates B1: 07/11/1993; B2: 30/07/1998; B3: 28/07/2003; B4: 30/06/2008. C) The 5- year series at Riou Archipelago; dates C1: 20/06/2001; C2 05/01/2006.

The present study addresses the population dynamics over decades of ten structural sessile species (sponges and anthozoans) dwelling on coralligenous outcrops (∼25 m depth) in two areas in the NW Mediterranean Sea: the Riou Archipelago (Provence coast, SE France) and the Medes Islands (Catalan coast, NE Spain). This study is unique due to its resolution, as it was based on the fates of annual censuses of identified specimens over periods of 25-, 15-, and 5- years. The photographic long-term records (see Fig. 1) from this study provide an exceptional opportunity to characterize the demographic traits of these marine temperate species over time. First, we provide demographic data (mortality, recruitment, growth, and turnover rates) for ten benthic species over a long period to document their natural variation and to identify certain relevant events (e.g. peaks of mortality, pulses of recruitment) in these populations. Second, based on our growth data we extend our demographic approach to project the longevity of these species. Finally, we synthesize the results obtained in this study by comparing the 10 species and relating their life-history traits to better understand the dynamics of coralligenous outcrops.

## Materials and Methods

### Study sites and species

The study was carried out in two main areas of the NW Mediterranean Sea: the Riou Archipelago (43°10′40″N, 5°23′50″E, SE France) and the Medes Island Marine Protected Area (42°3′N, 3°13′E, NE Spain) (Fig. S1). In these two areas (400 km apart), 4 study sites harboring coralligenous outcrops were selected. These communities are found in dim light conditions. Specifically, we studied 3 open submarine caves in the Riou Archipelago and one north-facing sublittoral wall in the Medes Islands. No specific permits were required for the described field studies. No specific permits were required for the described field studies. The two study areas: the Riou Archipelago and the Medes Islands do not require any specific permission. The locations are not privately-owned. This study did not involve endangered or protected species. Moreover, we did not perform any disturbance to species during our fieldwork. Our data was based on the analysis of images and this approach is a non destructive technique to study marine benthic communities.

We chose ten species for this study: 6 sponges (*Aplysina cavernicola*, *Chondrosia reniformis*, *Haliclona fulva*, *Scalarispongia (Cacospongia) scalaris*, *Petrosia ficiformis*, and *Spirastrella cunctatrix*) and 4 anthozoans (*Alcyonium acaule*, *Caryophyllia inornata*, *Corallium rubrum*, and *Leptopsammia pruvoti*). See Table 1 for general characteristics of the ten species. These species were selected according to fourth distinct criteria. First, they contribute greatly to the benthic seascape in coralligenous outcrops of the NW Mediterranean Sea, over a wide depth range (from 10 to 60 m depth). Second, they are macroscopic and relatively easy to identify. Third, they exhibit different growth forms, such as encrusting, cup, tree, and massive forms, which present different ecological strategies in occupying space on rocky benthic habitats [27], [28]. Finally, they are representative of a community structured by long-lived components for which population ecology is poorly understood.

**Table 1:** The ten species under long-term study in Mediterranean coralligenous communities.

Species	*	**	Morphological description	Reproduction type	Geographic area and depths	Photographic Series	Half-life (year)
*Aplysina cavernicola*	S	M	Yellow-massive sponge, with irregular disposed digitate extensions	Gonochoric, oviparous, unknown larvae^1^	Mediterranean Sea. Dimly lit habitats^3^	5 yr	not reached yet
*Chondrosia reniformis*	S	M	Spoted sponge with dark-brown and white spots, lobate with firm consistency	Gonochoric, oviparous, unknown larvae^1^	Mediterranean & Atlantic. Dimly lit habitats. Depth range: up to 60 m^3^	15 yr	not reached yet
*Haliclona fulva*	S	E	Orange encrusting sponge, oscula at the end of short oscular chimeneys	Hermaphroditic, viviparous, swimming larvae^1^	Mediterranean & Atlantic coast (Guinea & Canary Islands). Dimly lit habitats^3^	5 yr	not reached yet
*Petrosia ficiformis*	S	M	From ficiform shaped to irregularly globular forms, with fused globes, red color due to cyanobacteria	Gonochoric, oviparous, swimming larvae^1^	Mediterranean Sea & Atlantic (Azores, Canary Islands, Cape Verd). Sublittoral vertical walls, caves, and crevices. Depth range: 10–40 m^3^	15 yr	not reached yet
*Scalarispongia* *(Cacospongia) scalaris*	S	M	Light to medium grey, massive sponge, with an unarmoured and conulose surface.	Unknown, viviparous, swimming larvae^1^	Mediterranean Sea & Atlantic (Portugal, Azores, Canary Islands). Dimly lit habitats (sublittoral vertical walls, caves, and crevices). Depth range: up to 60 m depth^3^	25 yr	not reached yet
*Spirastrella cunctatrix*	S	E	Soft-orange encrusting sponge, with vein-like surface canals	Supposedly gonochoric, oviparous, supposedly creeping larvae^1^	Mediterranean Sea. Dimly lit habitats^3^	15 yr	not reached yet
*Alcyonium acaule*	A	T	Red colonial alcyonacean with a massive treelike growth form	Gonochoric, surface brooder, planula larvae, limited dispersal capacity^2^	NW Mediterranean Sea. Dimly lit habitats (vertical and horizontal rocky bottoms). Depth range: 10–45 m^2^^,^ 4	15 yr	10 yr
*Caryophyllia inornata*	A	C	Solitary, azooxanthellate cup- coral	-	Mediterranean Sea and Atlantic (Manche, Azores, Canary Islands). Dimly lit habitats (vertical or overhanging rockfaces and sea caves). Depth range: from surface to 100 m^8^	25 yr	10 yr
*Corallium rubrum*	A	T	Red colonial octocorallian with arborecent growth form	Gonochoric, brooder, planula larvae, limited dispersal capacity^5^	Mostly W Mediterranean Sea but also occurs E Mediterranean and African Atlantic coast. Dimly lit habitats (caves, vertical cliffs and overhangs). Depth range: 10–800 m^6^	25 yr	not reached yet
*Leptopsammia pruvoti*	A	C	Solitary, azooxanthellate scleractinian coral	Gonochoric, brooder, planula larvae^7^	Mediterranean Sea and along Atlantic coast from Portugal to southern England. Dimly lit habitats (under overhands and sea caves). Depth range: from surface to 70 m depth^8^	5 yr	16 yr

1 A. Ereskovsky 1 (*pers. comm.*);

2 N. Teixidó (unpubl. data);

3 MJ. Uriz (*pers. comm.*),

4 Gili *et al.* (1984),

5 Vighi (1972),

6 Zibrowius et al. (1984),

7 Goffredo et al. (2006),

8 Zibrowius (1980).

Species are ordered as a function of their group.

*Group: S: sponges; A: anthozoans.

**Growth forms: E encrusting; T tree; C cup: M mound.

### Photographic series over 25-, 15-, and 5- years

At each surveyed site, permanent plots were photographically monitored annually over 25- (from 1981 to 2006), 15- (from 1993 to 2008), and 5- years (from 2001 to 2006). The 25- year photographic series was obtained at the Riou Archipelago (Riou Sud cave). This series corresponded to photographs of ten experimental panels (20*20 cm each), made of local limestone, at a depth of 27 m on the eastern lateral wall of a submarine cave 20 m from the entrance. This experiment began in 1969 as part of a project aimed at studying the colonization of sublittoral hard substrates under natural conditions [29]. The 15- year photographic series was obtained at one site in the Medes Island area, where 10 permanent plots set on a vertical wall at 18–20 m depths were surveyed. Finally, the 5- year photographic series was obtained at three sites from the Riou Archipelago area. At each site, ten permanent plots were set up at depths between 20–24 m on the walls of three caves. For the 15- and 5- year series, the permanent plots were installed haphazardly with in an area of 30 m^2^ in the sampling areas. All images were taken with Nikonos cameras (Nikon, Tokyo, Japan) equipped with UW 28 mm and close-up lenses from 1981 to 2005. From 2006 onward, a Nikon D70S digital SLR camera fitted with a Nikkor 20 mm DX lens (3000 * 2000 pixel resolution), and Subal D70S housing was used. Lighting was provided by two electronic strobes (Nikonos SB 105 and Sea & Sea strobes). Each 35-mm frame recorded an area of 310 cm^2^ in size, and each digital frame recorded an area of 575 cm^2^. We restricted our analyses to the 310 cm^2^ initial area, despite the larger area covered by the digital camera to ensure the same measurements over time. Approximately 900 photographs were analyzed. For all of the photographic series analyzed, the photograph frames were treated as statistical replicates in a repeated measures design testing for differences over time.

### Demographic analysis

Demographic parameters were quantified using digital images, which were obtained for the color slides, by scanning the originals images (300 dpi, 1632×1080 pixel resolution). All individuals of the 10 target species were individually labeled in Adobe Photoshop 7.0 (© Adobe) and censused annually until death was confirmed in successive censuses. Three of the species (*S. scalaris, C. inornata, and C. rubrum*) have been studied since 1981 (25-year of data); four species (*C. reniformis, P. ficiformis, and S. cunctatrix and A. acaule*) since 1993 (15-year of data) and three species (*A.cavernicola*, *H. fulva* and *L. pruvoti*) since 2001 (5-year of data). A total of 180, 148, and 343 individuals present at the beginning of the three series were individually identified and followed over 25-, 15- and 5- years, respectively, to record their fates: mortality, recruitment via larvae, growth, and longevity. These analyses generated more than 8,000 observations.

### Mortality

Population dynamics were quantified by recording whether the specimens present at the initiation of the censuses were alive or dead at the end of the census period. We considered whole mortality to occur in the year when the entire specimen had completely disappeared from the analyzed plot. We calculated the annual relative mortality rate as follows:
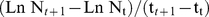
(1)where N refers to the number, and t_t_ and t_t+1_ (with units in years), are the initial and final census dates, respectively [30], [31]. The time interval in equation (1) is one year.

### Recruitment

We estimated larval recruitment annually by monitoring the development of new specimens that were clearly not created by asexual events (e.g. clonal fragmentation) within the surveyed areas. As larval recruits were not easily identified at early stages, we scored new recruits via larvae only if they reached a minimal size (approximately 1 mm^2^) and survived for more than one year. This approach gave a conservative estimation of recruitment for the ten species analyzed. Newly established individuals were individually labeled and censused as long as they remained alive. Recruitment was standardized to the initial adult population size for each species.

### Growth rate and estimated longevity

The growth rate for each colony was calculated as follows:
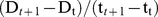
(2)where D refers to the diameter and t*_t+1_* and t_t_ are the initial and final census dates, respectively. The time interval considered for growth measurements was 5 years. The diameter of sponge and scleractinian colonies was calculated using the program CPCe 3.4 [32]. Growth of *A. acaule* and *C. rubrum*, which exhibit a tree-like form, was measured as any change in the development of branching. Growth measurements were performed only for the specimens (187 individuals) that were alive during the entire study period (25-, 15-, and 5-years).

To estimate longevity, four annual growth rate estimates were generated for each species based on the mean, upper quartile, upper decile, and maximum of long-term average values observed in the population. For *C. rubrum*, we used published growth data based on skeletal growth rings to estimate longevity due to the high precision of this technique [33], [34]. Four separate estimates of longevity were generated by dividing the diameter of the largest benthic species encountered (in the literature or in this study) by the mean, upper quartile, upper decile, and maximum growth rates obtained in this study. Our use of four descriptors (mean, upper quartile and decile, and maximum growth rates) to estimate the longevity of each species reflects a general consensus regarding the difficulty of performing demographic analyses in clonal organisms due to the fact that they exhibit the potential for indeterminate growth and the capacity to undergo both fusion and fission and to recover from partial mortality [27], [35], [36]. Longevity data for *A. acaule* are not presented in this study because of the very slow growth patterns of this species, causing it to exhibit extreme ages.

### Turnover rate calculations

We followed standard procedures applied in forest communities to calculate population change [37], [38], [39]. We consider this calculus a good approximation to calculate instantaneous measures of change per unit of the marine benthic species population. Let the census interval be *t*, and population size at time *t* and time *t+1* be *n_t_* and *n_t+1_*. The number of survivors at time *t+1* is *S_t+1_*, so the number of recruits is *n_t+1_−S_t+1_*.

Net change was calculated as follows:
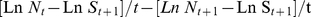
(3)In this study, the time interval in equation (3) is one year.

### Life histories of species

We compiled the most relevant life-history characteristics of the 10 species. Each species was ranked from 1 to 4 based on mortality and recruitment rates, maximum growth rates, % of no growth or negative growth, longevity, and turnover rates obtained throughout this study.

### Statistical analyses

A non-parametric analysis of variance, PERMANOVA [40], was used to examine the temporal trends among the different demographic parameters obtained. For mortality and recruitment, analyses were performed to test for differences among years (1-way PERMANOVA) and between time intervals and species (2-way PERMANOVA). For the latter analysis, annual censuses were pooled over intervals of five years. Thus, the 25-, 15- and 5-year series contained 5, 3, and 1 interval(s), respectively. Species was analyzed as a fixed factor, whereas years and time intervals were random. In addition, 1-way PERMANOVA was applied to test for differences in growth at the beginning and the end of the studied period. Finally, 1-way PERMANOVA was performed to calculate changes in turnover rates across time intervals. Differences between samples were quantified using Euclidean distances and 4999 unrestricted random permutations of the raw data were performed [41]. Pair-wise comparisons for all combinations of Species×Time intervals were also carried out using t-tests and 9999 permutations of the raw data. The analyses were computed using the program Primer v6 with the PERMANOVA + add-on package [42].

## Results

### Long-term trends in mortality rates

Analyses of the photoquadrats identified (i) 180 initial individuals that could be tracked between 1981 and 2006 (25-year data) belonging to *Scalarispongia scalaris* (n = 28 specimens), *Caryophyllia inornata* (n = 100), *and Corallium rubrum* (n = 52); (ii) 148 individuals between 1993 and 2008 (15-year data) belonging to *Chondrosia reniformis* (n = 16), *Petrosia ficiformis* (n = 30), *Spirastrella cunctatrix* (n = 52) *and Alcyonium acaule* (n = 50); and (iii) 343 individuals between 2001 and 2006 (5-year data) belonging to *Aplysina cavernicola* (n = 85), *Haliclona fulva* (n = 71), and *Leptopsammia pruvoti* (n = 187).

**Figure 2:**
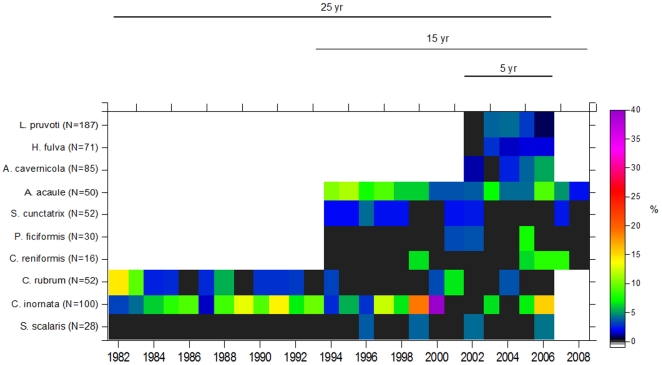
Long-term trends in the mortality rates of the ten species in coralligenous communities. Data include the annual mortality rate (%) for each species from 1982–2008 (25-, 15-, and 5- year time series). The number of individuals at the beginning of the study is shown in parentheses. Note that black areas indicate zero mortality, whereas white areas indicate no data.

Overall, the mean mortality rate was low with values of 3.4%±0.4 being obtained for all ten species (n = 671) (Fig. 2). Nearly all species in each census had rates below 5% (75% of instances). Mortality rates did not differ significantly among years (F_24,117_ = 0.41, p>0.05) and were fairly consistent between time intervals (five-year period) (F_4,112_ = 0.25, p>0.05) (see Table S1 for 2-way PERMANOVA test). There was no significant interaction found between species and the time intervals (F_16,112_ = 1.6, p>0.05). However, mortality patterns differed significantly among species (F_9,112_ = 5.3, p = 0.004). Considering the species term, pair-wise comparisons showed significant differences among species. For example, the scleractinian *C. inornata* and the alcyonacea *A. acaule* showed the highest mean rates of mortality (9.2%±2.3 SE and 6.8%±0.8, respectively) and differed significantly from the sponges *S. scalaris*, *C. reniformis*, and *P. ficiformis*, which exhibited much lower mortality rates (0.5%±0.3 SE, 2.2%±1.3 SE, and 1.1%±0.6 SE, respectively). *C. inornata* showed the highest mortality rate, with a value of 40% in 1999–2000. Conversely, the lowest mortality rate was zero, observed in 45% of instances, with the most extreme being for the massive sponge *S. scalaris*, in which only 4 out of 28 individuals died in different years during the 25-year of investigation. The red coral *C. rubrum* showed an intermediate level of mortality of 2.7%±0.7 SE. Overall, these values indicate that a half-life (time until 50% of initial individuals are dead) was not reached for the majority of the species, for example *C. rubrum* and *S. scalaris* (Table 1).

### Long term trends in recruitment patterns

**Figure 3:**
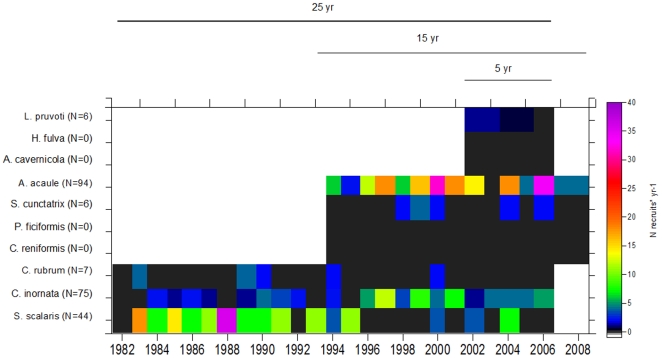
Long-term trends in recruitment of the ten species in coralligenous communities. Recruitment was measured as the number of recruits * yr^−1^ for each species from 1982–2008 (25-, 15-, and 5- year time series) and was transformed prior to analyses. The total number of recruits for each species during the study period is shown in parentheses. Note that black areas indicate zero recruitment, whereas white areas indicate no data.

Recruitment was only observed for 6 out of the 10 surveyed species over the study period (Fig. 3). A total of 232 recruits were recorded mainly belonging to three species *A. acaule* (40%), *C. inornata* (32%), and *S. scalaris* (19%). Between 0 and 7 recruits were observed for the other species over the study period. Therefore, larval recruitment was low, oscillating between 0 (in 58% of instances) and 17 recruits (in 0.5% of instances) (Fig. 3). No recruits were recorded for *C. reniformis* and *P. ficiformis* (during 15-years) or for *A. cavernicola* and *H. fulva* (during 5-years) (Fig. 3). The mean recruitment rate was 3.1±0.5 SE recruits yr^−1^ for the ten species and annual censuses. The differences in recruitment patterns were not significant among years (F_24,117_ = 0.62 p>0.05) but differed significantly among species (F_4,112_ = 4.5, p = 0.01) and for the interaction term between species and time intervals (F_16,112_ = 2.5, p = 0.003) (see Table S2 for 2-way PERMANOVA test). For the sponge *S. scalaris*, recruitment was 9.2±1.6 SE and 14.3±2.0 SE recruits year^−1^ over the first and second intervals, respectively. Similar trends were found for the scleractinian *C. inornata* (with 6.8±1.0 SE and 1.5±0.7 SE recruits year^−1^) and the alcyonacea *A acaule* (with 18.0±1.6 SE and 14.0±2.2 SE recruits year^−1^) over the fourth and fifth intervals, respectively. Mortality among the recruits was high, and approximately 50% of the recruits died during the first 5 years after the first observations. Interestingly, more than 60% of *A. acaule* recruits died during the first two years. Conversely, recruit mortality was by far the lowest for *S. scalaris* (27% after 25-years), and they survived for a longer time, with a mean age of 13.8 yr±1.07 being observed.

### Growth patterns

Changes in both surface area and the number of branches of 187 specimens belonging to the 10 species monitored over 25-, 15-, and 5- years are presented in Fig. S2. The data on surface area and branching patterns represent net growth measurements for each census over the study period. Overall, we observed low and asynchronous growth in all specimens across the ten species analyzed (Fig. S2, Table 2). In 50% of instances, there was negative or no growth detected for the ten species, corroborating the existence of a low growth pattern (Table 2). However, this pattern varied among species. Based on surface area, only the sponge *S. scalaris* out of 8 species studied exhibited a significant change in growth from the beginning (mean value of 140 mm^2^±31.4) to the end (mean value of 2203.9 mm^2^±415) of the study period (25-years) (interaction term F_7, 267_ = 4.4, p<0.0001, pair-wise comparison p<0.0001). Of the two branching species, only the red coral *C. rubrum* (interaction term F_1, 86_ = 12.8, p<0.001, pair-wise comparison p<0.0001) exhibited a significant increase in the number of branches, from colonies with less than one branch at the beginning of the study period to colonies with a mean value of 4.5±0.5 branches after 25 years. The other species did not show any statistically conspicuous growth from the beginning to the end of the investigation periods. Extreme cases were observed for the sponges *C. reniformis* and *P. ficiformis*, which exhibited a decrease of surface area, and the alcyonacea species *A. acaule*, *which* showed a mean increment of 1±0.4 fingers after 15 years (n = 19) (Table 2).

**Table 2:** Growth of the ten species in Mediterranean coralligenous communities.

	N	Measured period	% 0 0r neg.	Max Diamterer or n branches	Growth rates (mm/yr) or n° branches/yr			
Species					Mean	Upper quartile	Upper decile	Max
*Leptopsammia pruvoti*	12	5 & 25 yr	39%	17 mm^c^	0.70	1.18	1.96	3.33
*Haliclona fulva*	19	5 yr	37%	260 mm^a^	6.63	7.9	12.9	19.9
*Aplysina cavernicola*	26	5 & 25 yr	28%	220 mm^a^	5.0	7.7	15.0	26.6
*Alcyonium acaule* *	19	15 yr	82%	45 fingers^a^	-	-	-	-
*Spirastrella cunctatrix*	29	15 yr	47%	110 mm^a^	neg	5.2	8.1	17.6
*Petrosia ficiformis*	12	15 yr	41%	252 mm^a^	neg	6.2	7.2	8.0
*Chondrosia reniformis*	9	15 yr	48%	180 mm^a^	neg	6.1	7.3	9.8
*Corallium rubrum*	27	25 yr	76%	30 mm^b^	0.15^b^	0.17^b^	0.23^b^	0.44^b^
*Caryophyllia inornata*	12	25 yr	52%	20 mm^c^	0.68	1.3	1.66	2.6
*Scalarispongia scalaris*	27	25 yr	30%	180 mm^a^	6.06	8.45	10.8	11.8

Notes:

a : N. Teixidó (unpublished data),

b : Torrents (2007),

c : Zibrowius (1980).

*Growth data for *Alcyonium acaule* is not presented due to the extreme low growth values.

Annual growth rate estimates (diameter or n° branches) were generated for each species based on mean, upper quartile (highest 75% of data), upper decile (highest 90% of data), and maximum values. Data also include the percentage of individuals showing 0 or negative growth over each census (% 0 or neg.). Max Diameter indicates the largest colonies encountered in the field or literature.

### Estimated longevities

**Figure 4:**
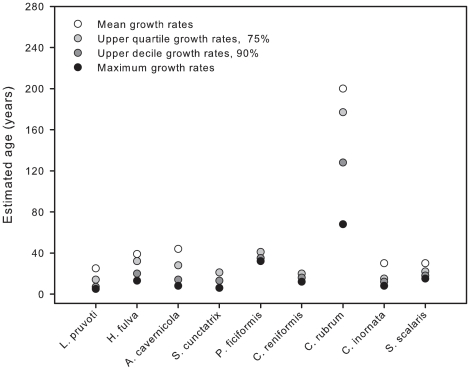
Maximum longevity data for the 10 benthic species. These data are based on the largest size encountered and growth rate measurements (mean, upper quartile, upper decile and maximum).

Considering the 10 species together, the mean estimated longevity of individuals was 62±28 years, with maximum and minimum values of 200 and 25 years, respectively (Fig. 4). To provide an approximation of the range of longevity, seven species showed intermediate longevities (25–45 years) and one species was much longer lived than all the others (*C. rubrum* approximately 200 years). Based on the maximum (range from 5 to 68 years) and mean (from 25 to 200 years) longevity estimations (Fig. 4), it is reasonable to conclude, that very large individuals of all the studied species are at least 20–70 year old.

### Turnover

**Figure 5:**
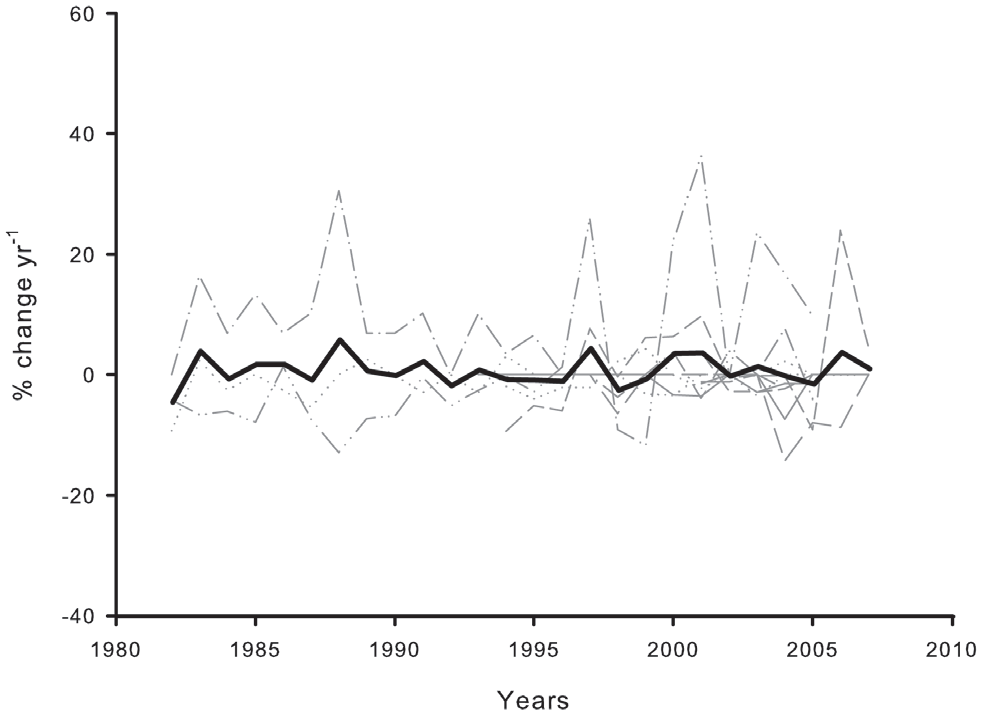
Rates of population change through time for the ten benthic species. The black line represents the mean.

We now summarize the population change patterns for the ten benthic species based on mortality and recruitment over 25-, 15-, and 5- years (Fig. 5). The mean turnover pattern for all of the species, showed slow rates over time (0.80±0.5% yr^−1^) and it was not statistically conspicuous over the investigated time intervals (F_4,24_ = 0.72, p>0.05). As an example, the coral *C. rubrum* and both sponges *C. reniformis* and *P. ficiformis* exhibited no change in 45% and >70% of instances over the 25- and 15 -year periods, respectively.

### Life histories of species

Table 3 summarizes the most relevant life-history characteristics analyzed throughout this study. Overall, the species exhibit a gradient of common demographic and life-history patterns of low mortality, recruitment and grow, and moderate to high longevity. The majority of the species (n = 7) exhibiting different growth forms showed low mortality, recruitment, and turnover rates, low growth and moderate longevity. However, the scleractinian *C. inornata* and the alcyonacea *A. acaule* exhibited the highest mortality and recruitment rates and high rates of turnover. Interestingly, *C. rubrum* presented the highest longevity and the highest proportion of no growth over time.

**Table 3:** A summary of the demographic and life-history characteristics of the ten species under study.

Characteristics	*Alcyonium acaule*	*Caryophyllia inornata*	*Spirastrella cunctatrix*	*Scalarispongia scalaris*	*Corallium rubrum*	*Haliclona fulva*	*Leptopsammia pruvoti*	*Aplysina cavernicola*	*Chondrosia reniformis*	*Petrosia ficiformis*	Table/Figure
Group	A	A	S	S	A	S	A	S	S	S	
Growth forms	T	C	E	M	T	E	C	M	M	M	
Mortality	1	1	3	4	4	3	3	3	3	4	Figure 1
Recruitment	1	1	2	1	2	4	3	4	4	4	Figure 2
Growth (max)	-	2	1	2	4	1	2	1	2	2	Table 2
% 0 or neg. Growth	1	2	2	3	1	3	2	2	2	2	Table 2
Longevity		4	4	3	1	3	4	4	3	2	Figure 3
Turnover	2	2	3	2	4	4	4	4	4	4	Figure 4
**Mean Rank**	**1.25**	**2**	**2.5**	**2.5**	**2.6**	**3**	**3**	**3**	**3**	**3**	

Species are ordered from lowest (4) to highest (1) rank values. Group: S sponges; A anthozoans. Growth forms: E encrusting; T tree; C cup; M mound.

## Discussion

Marine ecosystems are facing a global decline of marine sessile species in many benthic communities due to the increase of disturbance regimes linked to global change [43], [3], [44]. Under this scenario, scientists and managers are putting forth the questions of the ecosystems' capacity to recover after natural and human-induced disturbances and how their populations will change. Therefore, basic knowledge regarding the demographic processes of sessile marine species at relevant spatial and temporal scales is becoming crucial, especially for long-lived species. The present long-term study quantitatively revealed a common life-history pattern among the ten studied species, despite the fact that they display different morphological forms, in which low mortality was accompanied by infrequent recruitment events, low growth rates of adults, and estimated life-spans covering from decades to centuries (Fig. 2, 3, 4). These results significantly expand our knowledge related to the population dynamics of coralligenous Mediterranean species, and they are consistent with previous studies of long-lived structural species [45], [46], [47], [48]. It is important to note that, rather than clearly distinguishable types of life histories, a continuum of demographic patterns was found within the K- selection traits of the studied species (Table 3) [49], [50]. Moreover, the low to moderate turnover rates (mean value of 0.80% yr^−1^) observed among species corroborated their low dynamics and persistence (Fig. 5). These demographic traits correspond to those expected based on theory of life-history evolution for species that grow in stable environmental conditions [19]. Age estimations provide (mean estimated age from 25–200 years) a first approximation of how ages are distributed among structural species (Fig. 4) and underscore the importance of age demographics in the ecological structure and function of these outcrops. This study represents a pioneer effort to better understand species' life-history traits over long temporal scales to comprehend the natural variability of demographic process and evaluate ecological significance in terms of community structure and dynamics.

The mortality rates for all 10 studied species were low (3.4% yr^−1^ for all species combined), did not change during the study period, and were 0 in 45% of the cases (Fig. 2). Moreover, none of the populations that we originally followed for 25-, 15-, and 5-years disappeared. These results suggest that natural mortality in the adult population is low, thus leading to high persistence of the community. Mortality rates in other long-lived benthic species have also been reported to be low but depend greatly on species (see below) and size class [51], [46], [26], [52]. The mortality rates for the scleractinian *Caryophyllia inornata* (mean value 9.2±2.3% yr^−1^) and the alcyonacea *Alcyonium acaule* (mean value 6.8±0.8% yr^−1^) were significantly high (Fig. 2). Moreover, the former species exhibited peaks of mortality as high as 14 and 40% yr^−1^ (1991, 1999, 2000, 2006). This may indicate that variability among species and over short time scales is also important. The mortality rates of these species were high relative to their slow growth and moderate recruitment (Fig. 2, Fig. 3, Table 2). Interestingly, these two species showed the highest recruitment among the 10 species with peaks as high as 32 recruits yr^−1^ for *A. acaule* (4-fold higher than the overall mean, see below). However, the survival among recruits was low (50 and 75% for *C. inornata* and *A. acaule*, respectively), and more than 60% of the recruits of *A. acaule* died during the first two years of observation. This is reinforced by the ∼10 years required for a surviving recruit of *A. acaule* to attain the size of a 1- finger- colony (largest size encountered in nature is an ∼45- finger- colony) (N.Teixidó, unpublished data). Adult mortality in long-lived species has been reported to be a key factor for population persistence because it will influence the time that a population can persist without recruitment [47], [17], [22], [52]. Additionally, Huston [53] suggested that high densities of long-lived individuals with low growth rates should arise only over relatively long periods of time under conditions of low mortality. Importantly, immediate and delayed mortality rates in extraordinary events, such as the mass mortality events of 1999 and 2003 in the NW Mediterranean Sea, have been reported to be higher than 10% and 48% of the initial population, respectively [16], [54], [18]. The ten studied species were among the species affected by these mortality outbreaks [55], [18]. Thus, the relevance of this study is that it provides natural mortality rates over a long temporal scale (decades) that can be contrasted with data obtained after unusual, -low frequency events.

Our surveys performed using 25-, 15-, and 5- year photographic series revealed that recruitment was generally low (mean value of 3.1±0.5 SE recruits yr^−1^) and did not differ among years, providing only a very small fraction of the new colonies needed to maintain population sizes (Fig. 3). Extreme cases were observed for the massive sponges *Chondrosia reniformis* and *Petrosia ficiformis* (15- years), *Aplysina cavernicola* and the encrusting species *Haliclona fulva* (5- years), for which no recruitment was observed over the study period. Moreover, even though the other six species showed discrete recruitment events, 50% of these new recruits did not survive 5 years after their detection. Therefore, it is relevant to emphasize that despite the importance of recruitment processes, the survivorship of new individuals over time is crucial for the establishment and maintenance of populations [28], [56], [57]. Hughes and Tanner [26] emphasize the distinction of two types of mechanisms to explain the possible causes of recruitment failure: i) factors related to competition for substrate (filamentous and fast-growing macroalgae or other invertebrates), predation, and physical disturbances; and ii) factors related to the decrease of the larval pool or lower fecundities associated with positive seawater temperature anomalies [58], [59], [60]. The relative importance of recruitment vs. mortality in determining the viability of sessile marine species can be obtained from demographic studies using matrix transition probabilities to explore the sensibility of population growth rates [22], [48]. Our recruitment results suggest that this process probably does not strongly influence the abundance of the adult populations. Within this context, the 10 species studied presented life-history traits characteristic of organisms with a high life expectancy within the bet-hedging theory: low and variable recruitment but high and constant adult survival [19].

Longevity and slow growth are not unknown in coralligenous species. In this study, we demonstrate the existence of a continuum of age estimates (∼50 yr) (Fig. 4), which provides a solid overview of the long life spans of these species but also shows the among-species variation in longevity. This existence of such a continuum in longevity and life histories in these species despite showing different morphological forms suggests that they may have experienced common evolutionary routes. Accurate estimates of life spans are crucial for understanding evolutionary relationships and population and community dynamics. In addition, interest in the conservation and protection of coralligenous outcrops, resulting from their long life spans, recognition of their ecological importance, and threats posed by fishing practices, habitat destruction, recreational diving, and invasive species has increased considerably. The long life spans of the ten species shown here reinforce the need for further protection of this community [61]. The longevity of *Corallium rubrum* challenges the concept that this species is renewable in the context of fisheries management [13], [62]. In addition, damage to these sponges and octocoral species has far-reaching implications for biodiversity, ecosystem structure and function.

We found that mean turnover rates of coralligenous species were low over the time period studied (Fig. 5). Overall, this result indicated that there was a synchronized response of the two parameters, with recruitment occasionally exceeding mortality due to the recruits of *A. acaule* and *C. inornata*. Species turnover is an emergent property of underlying structural and dynamic community processes [63], [64], [65]. Coralligenous outcrops are driven mainly by endogenous growth, in which energy supplies and biological interactions, such as competition for space, are important drivers [66], [13], [12]. However, exogenous disturbance events, such high positive temperature anomalies or severe storms (e.g. an unusual and dramatic storm in December 2008 with waves 12 m high), have affected these communities during the last decades, and climatic scenarios predict an increase of environmental variability as a result of more frequent extreme climatic events [67]. The question behind it is, whether catastrophic disturbances occur frequently and synchronously enough to generate large-scale lags of recruitment following mortality, or whether they are so rare and random that, instead, pulses of recruitment lead to pulses of mortality. Adaptation of species to disturbance depends on coupling between disturbance frequencies and lifetimes and is evolutionarily important [68], [69]. If these dramatic disturbances occur frequently and synchronously enough to generate pulses of mortality with lags in recruitment, then this poses serious questions about the consequences for biodiversity maintenance, the structure and functioning of these communities, and their resilience in the face of ongoing climate change.

We would like to point out that it may not be ideal to establish the late nineteenth- or early twentieth- century as a baseline concerning population and community dynamics in the Mediterranean Sea, because the whole system may be undergoing a shift. For instance, overfishing has severely reduced the number of large-sized individuals [70], [71], and several coral species from the Pacific Ocean and the Mediterranean Sea have been extensively harvested for jewelry [72], [13], [62]. Therefore, we hypothesize that this reduction of large-sized organisms has ultimately led to a shift in the size distribution of sessile invertebrates, with repercussions for their longevity and persistence, in particular, and in their population dynamics in general. However, we believe that our findings obtained over decades and different geographical areas provide consistent patterns regarding the natural variability of demographic parameters and life-history traits.

In the present study, 25-, 15-, and 5- year photographic records from two localities in the NW Mediterranean Sea provided baseline data on the demographic processes associated with ten sessile species (sponges and anthozoans), that are important structural components of coralligenous outcrops. The results presented here provide quantitative evidence of the natural variability in demographic traits previously attributed to these types of species over long time periods. In addition, this study has indicated critical life-history traits and ecological factors that may be important for the dynamics of coralligenous outcrops. Insights gained from demographic data underscore the value of annual surveys and measurements of individuals. Applying this approach to more coralligenous species, from multiple sites and over time, is likely to greatly extend our ability to model the dynamics of these complex, long-lived and resilient sublittoral benthic communities. Our findings are highly relevant for any mathematical model of population and community dynamics. Determining demographic parameters of species is fundamental to understand and predict the present and future dynamics of individuals, populations, or communities [73].

## Supporting Information

Figure S1 **Map of the study sites in the NW Mediterranean Sea.** Riou Archipelago (43°10′40″N, 5°23′50″E, SE France, 1 site with 25- year and 3 sites with 5- year of data) and La Pota de Llop, in the Medes Island Marine Protected Area (42°3′N, 3°13′E, NE Spain, 1 site with 15- year of data).
(TIF)

Figure S2 **Growth patterns of the ten species studied.** Changes in surface areas of the monitored specimens for the sponges *Aplysina cavernicola*, *Chondrosia reniformis*, *Haliclona fulva*, *Scalarispongia (Cacospongia) scalaris*, *Petrosia ficiformis*, and *Spirastrella cunctatrix* and the scleractinian species *Caryophyllia inornata* and *Leptopsammia pruvoti* or in number of branches for the octocoral species *Alcyonium acaule* and *Corallium rubrum*. The black line represents the mean ± SE.
(TIF)

Table S1 **Non-parametric univariate analysis of variance (PERMANOVA) of mortality rates based on Euclidean distances for annual mortality rates (%) of the 10 species.**
(DOC)

Table S2 Non-parametric univariate analysis of variance (PERMANOVA) of recruits based on Euclidean distances for the number of recruits * yr^−1^ of the 10 species.
(DOC)
