# Supplementary figures and images for: Low Dynamics, High Longevity and Persistence of Sessile Structural Species Dwelling on Mediterranean Coralligenous Outcrops
Source: PLoS One. 2011 Aug 24;6(8):e23744. doi: 10.1371/journal.pone.0023744 (PMC3161055; doi:10.1371/journal.pone.0023744)

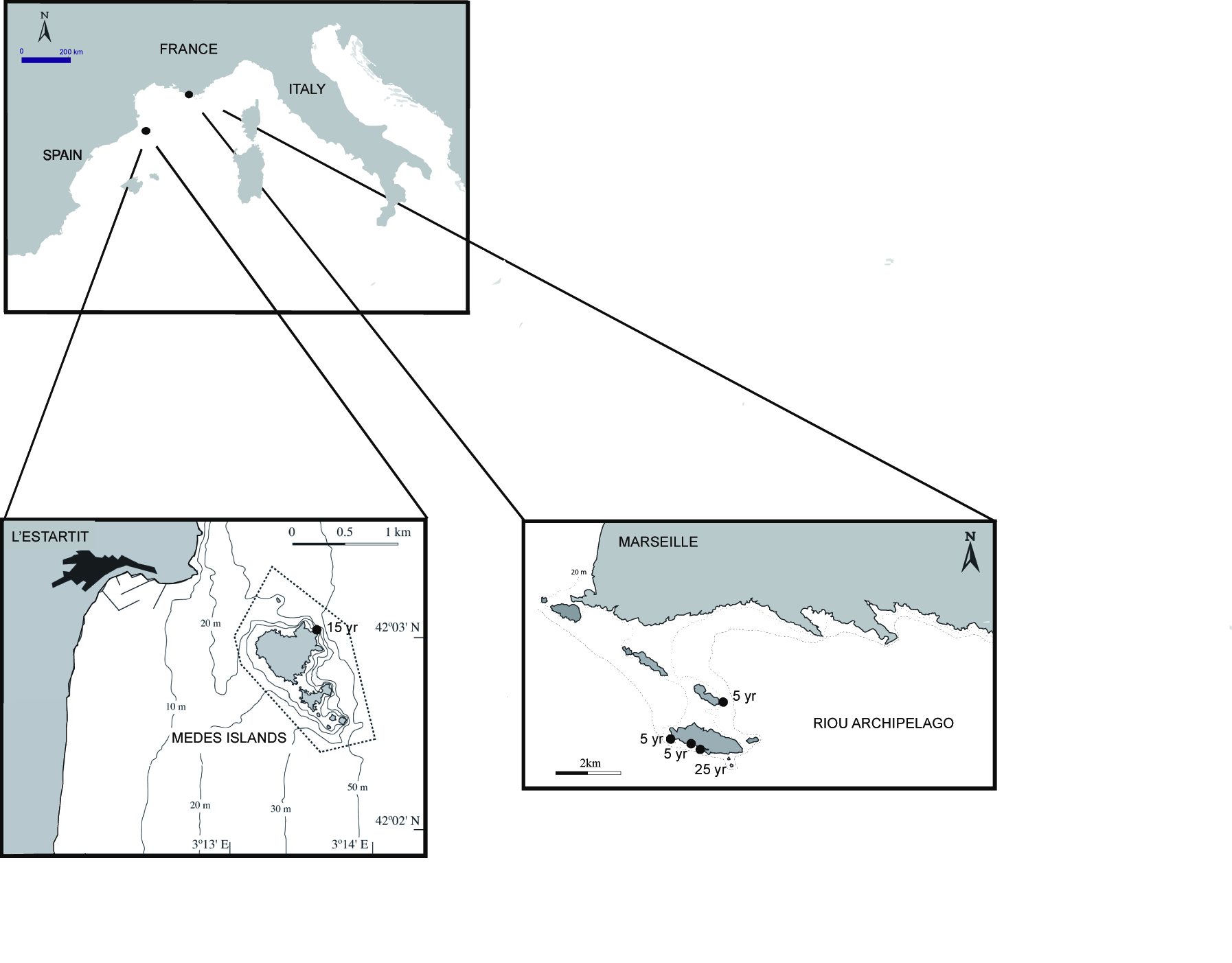

Supplement: Figure S1 — Map of the study sites in the NW Mediterranean Sea. Riou Archipelago (43°10′40″N, 5°23′50″E, SE France, 1 site with 25- year and 3 sites with 5- year of data) and La Pota de Llop, in the Medes Island Marine Protected Area (42°3′N, 3°13′E, NE Spain, 1 site with 15- year of data). (TIF) [file pone.0023744.s001.tif]

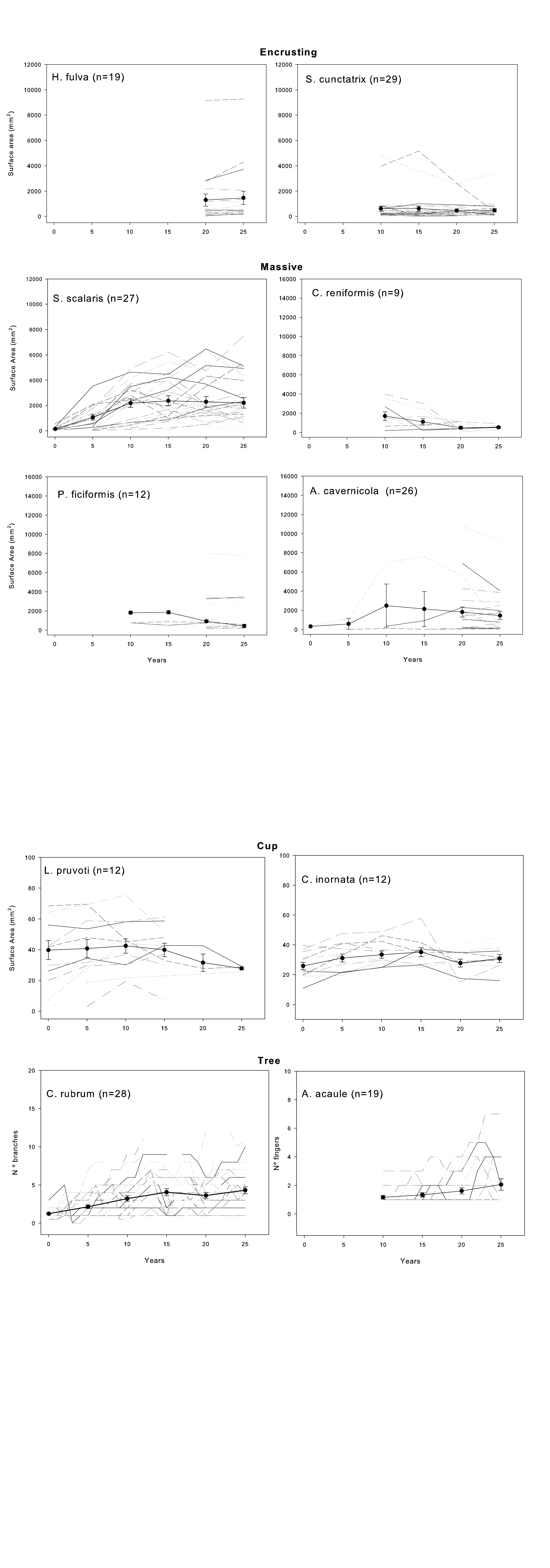

Supplement: Figure S2 — Growth patterns of the ten species studied. Changes in surface areas of the monitored specimens for the sponges Aplysina cavernicola, Chondrosia reniformis, Haliclona fulva, Scalarispongia (Cacospongia) scalaris, Petrosia ficiformis, and Spirastrella cunctatrix and the scleractinian species Caryophyllia inornata and Leptopsammia pruvoti or in number of branches for the octocoral species Alcyonium acaule and Corallium rubrum. The black line represents the mean ± SE. (TIF) [file pone.0023744.s002.tif]
