# Supplementary material for: Low Dynamics, High Longevity and Persistence of Sessile Structural Species Dwelling on Mediterranean Coralligenous Outcrops
Source: PLoS One. 2011 Aug 24;6(8):e23744. doi: 10.1371/journal.pone.0023744 (PMC3161055; doi:10.1371/journal.pone.0023744)
Supplement: Table S1 — Non-parametric univariate analysis of variance (PERMANOVA) of mortality rates based on Euclidean distances for annual mortality rates (%) of the 10 species. (DOC) [file pone.0023744.s003.doc]

**Table S1**. Non-parametric univariate analysis of variance (Permanova) on the basis of Euclidean distances for annual mortality rates (%) of the 10 species.

| **Source** | **df** | **SS** | **MS** | **F** | **p** |
| --- | --- | --- | --- | --- | --- |
| Species | 9 | 1400.2 | 155.58 | 5.3017 | 0.0042 |
| Time interval | 4 | 17.97 | 4.4924 | 0.25413 | 0.9046 |
| Species* Time interval | 16 | 470.03 | 29.377 | 1.6618 | 0.0703 |
| Residual | 112 | 1979.9 | 17.678 |  |  |
| Total | 141 | 3868.7 |  |  |  |
