# Supplementary material for: Low Dynamics, High Longevity and Persistence of Sessile Structural Species Dwelling on Mediterranean Coralligenous Outcrops
Source: PLoS One. 2011 Aug 24;6(8):e23744. doi: 10.1371/journal.pone.0023744 (PMC3161055; doi:10.1371/journal.pone.0023744)
Supplement: Table S2 — Non-parametric univariate analysis of variance (PERMANOVA) of recruits based on Euclidean distances for the number of recruits * yr−1 of the 10 species. (DOC) [file pone.0023744.s004.doc]

**Table S4**. Non-parametric univariate analysis of variance (PERMANOVA) on the basis of Euclidean distances for the number of recruits * yr^-1^ of the 10 species.

| Source | df | SS | MS | F | p |
| --- | --- | --- | --- | --- | --- |
| Species | 9 | 1996.7 | 221.86 | 4.4906 | 0.011 |
| Time interval | 4 | 191.75 | 47.937 | 2.4489 | 0.058 |
| Species* Time interval | 16 | 791.8 | 49.487 | 2.5281 | 0.003 |
| Residual | 112 | 2192.4 | 19.575 |  |  |
| Total | 141 | 5523. |  |  |  |
